# Diagnostic potential of circulating cell‐free microRNAs for community‐acquired pneumonia and pneumonia‐related sepsis

**DOI:** 10.1111/jcmm.15837

**Published:** 2020-09-11

**Authors:** Stefanie Hermann, Florian Brandes, Benedikt Kirchner, Dominik Buschmann, Melanie Borrmann, Matthias Klein, Stefan Kotschote, Michael Bonin, Marlene Reithmair, Ines Kaufmann, Gustav Schelling, Michael W. Pfaffl

**Affiliations:** ^1^ Division of Animal Physiology and Immunology School of Life Sciences Weihenstephan Technical University of Munich Freising Germany; ^2^ Department of Anesthesiology University Hospital Ludwig‐Maximilians‐University Munich Munich Germany; ^3^ Department of Neurology University Hospital Ludwig‐Maximilians‐University of Munich Munich Germany; ^4^ IMGM Laboratories GmbH Planegg Germany; ^5^ Institute of Human Genetics University Hospital Ludwig‐Maximilians‐University Munich Munich Germany; ^6^ Department of Anesthesia Klinikum Neuperlach Munich City Hospitals Munich Germany

**Keywords:** biomarker signature, cell‐free microRNAs, community‐acquired pneumonia, extracellular vesicles, sepsis, small RNA sequencing

## Abstract

Cell‐free microRNAs (miRNAs) are transferred in disease state including inflammatory lung diseases and are often packed into extracellular vesicles (EVs). To assess their suitability as biomarkers for community‐acquired pneumonia (CAP) and severe secondary complications such as sepsis, we studied patients with CAP (n = 30), sepsis (n = 65) and healthy volunteers (n = 47) subdivided into a training (n = 67) and a validation (n = 75) cohort. After precipitating crude EVs from sera, associated small RNA was profiled by next‐generation sequencing (NGS) and evaluated in multivariate analyses. A subset of the thereby identified biomarker candidates was validated both technically and additionally by reverse transcription quantitative real‐time PCR (RT‐qPCR). Differential gene expression (DGE) analysis revealed 29 differentially expressed miRNAs in CAP patients when compared to volunteers, and 25 miRNAs in patients with CAP, compared to those with sepsis. Sparse partial‐least discriminant analysis separated groups based on 12 miRNAs. Three miRNAs proved as a significant biomarker signature. While expression levels of miR‐1246 showed significant changes with an increase in overall disease severity from volunteers to CAP and to sepsis, miR‐193a‐5p and miR‐542‐3p differentiated patients with an infectious disease (CAP or sepsis) from volunteers. Cell‐free miRNAs are potentially novel biomarkers for CAP and may help to identify patients at risk for progress to sepsis, facilitating early intervention and treatment.

## 1 INTRODUCTION

According to the World Health Organization's global health estimates, lower respiratory infections are the fourth leading global cause of deaths and the most deadly communicable disease, causative for three million deaths worldwide in 2016.^1^ With the current SARS‐CoV‐2 pandemic, this fact has very recently been brought to worldwide attention. Data from a prospectively followed multicentre trial revealed an overall mortality of 17.3% for patients with CAP within an 18 months follow‐up.^2^ Despite the introduction of antibiotic therapies in the 1950s, pneumonia mortality has not decreased substantially,^3^ and sepsis, septic shock or acute pulmonary failure (eg acute respiratory distress syndrome, ARDS) are frequent secondary complications.^4^, ^5^

At present, initial pneumonia diagnosis is based on suggestive clinical features such as fever, shortness of breath, sputum production, cough and leukocytosis supplemented by evidence of pulmonary consolidation found in chest X‐rays or computed tomography (CT) if required in order to arrive at a final diagnosis. To improve management and treatment of pneumonia, supporting microbiological and virological tests from throat swabs, sputum or blood cultures might be indicated to identify the responsible pathogen(s) and to allow targeted antimicrobial or antiviral therapy. This can be supported by urine antigen tests, molecular assays, serology or bronchoscopy in selected cases. Blood biomarkers such as procalcitonin (PCT), C‐reactive protein (CRP), Interleukin‐6 (IL‐6), white blood cell count and lactate^6^, ^7^ are commonly used to differentiate between patients with pneumonia and individuals with pneumonia at risk for sepsis. Scoring systems including the *Confusion*,* Blood Urea*,* Respiratory Rate*,* Blood Pressure*,* Age ≥ 65* (CURB‐65) score ^8^ are applied in patients with pneumonia to simplify site‐of‐care decisions such as outpatient treatment *vs*. hospital and intensive care unit (ICU) admission and to facilitate the decision whether to prescribe antibiotics or not.^6^ However, the sensitivity and specificity of these scoring systems are limited.^9^ The reliable diagnosis of pneumonia can be a time‐consuming and complex process. It is particularly challenging in high‐risk groups^10^ such as the elderly or infants, which often present with atypical symptoms and are at an increased risk for sepsis or acute pulmonary failure as secondary complications.

At present, there are no valid and reliable biomarkers allowing an on‐site diagnosis and the identification of high‐risk patients.

Circulating EVs are a heterogeneous group of small‐sized membranous vesicles that are loaded with biomolecules, particularly proteins, lipids and diverse types of nucleic acids and are exchanged in cell‐cell signalling during various physiological and pathological processes.^11^ EV‐associated miRNAs are key regulators in the pathogenesis of infectious and non‐infectious pulmonary disorders.^12^, ^13^, ^14^, ^15^ Therefore, differential miRNA expression in EV samples from liquid biopsies may indicate the presence of an inflammatory lung disease and discriminate between different disease stages or even predict the course of the disease.^16^, ^17^ miRNAs might reflect progression from physical health, to mild and more severe forms of pneumonia.^18^ Additionally, EVs from sepsis patients contain miRNAs and messenger RNAs (mRNAs) related to disease‐associated pathways, such as inflammatory response, oxidative stress and cell cycle regulation.^19^

Therefore, extracellular miRNAs might be attractive diagnostic biomarkers for pulmonary inflammation and prognostic indicators for disease progression.

In the present study, we identified cell‐free miRNA biomarker candidates by high‐throughput small RNA sequencing (small RNA‐seq) to differentiate between patients with CAP and healthy volunteers, and to distinguish CAP patients from those with sepsis. The candidate biomarker signature was first technically validated by RT‐qPCR in the same training cohort of individuals and subsequently confirmed in a second, independent validation cohort.

## 2 METHODS

### 2.1 Study population

A total of 142 individuals were studied. Of those, 30 patients had CAP, 65 had sepsis and 47 healthy volunteers served as controls. The samples were subdivided into a training and a validation cohort. The training cohort included 67 individuals: twelve had CAP, 28 had sepsis (23 patients were in septic shock) and 27 were volunteers. The validation cohort consisted of 75 individuals: 18 with CAP, 37 with sepsis (30 patients were in septic shock) and 20 were volunteers. Patients with CAP were recruited consecutively from the emergency room of the Munich University academic centre located at the Ludwig‐Maximilians‐Universität (LMU) hospital. Sepsis patients were enlisted serially from the ICUs of the LMU hospital and the Neuperlach Community Hospital of Munich. Healthy volunteers were enrolled by advertisement and from hospital staff. Inclusion and exclusion criteria for the study subgroups are presented in detail in Table S1 of the online supplement.

CAP was defined as the presence of clinical symptoms such as fever, cough and respiratory impairment in addition to a CURB‐65 score for pneumonia severity ≥1^8^ and the demonstration of pulmonary infiltrates on chest X‐rays or CT scans according to the *Clinical Practice Criteria of the American Thoracic Society of America* (2019 version).^20^ Sepsis was defined in agreement to the updated consensus definition of the *Third International Task Force for Sepsis and Septic Shock* (Sepsis‐3)^21^ and diagnosed by using all available information (including imaging, antibiotic response and surgical findings). The final diagnosis of sepsis was made by experienced ICU and emergency room clinicians without prior knowledge of the results of the molecular studies.

The PCT plasma concentration was measured using a commercially available ELISA test (Brahms Procalcitonin Assay, Thermo Fisher Diagnostics GmbH, Hennigsdorf, Germany). IL‐6 levels, neutrophil gelatinase‐associated lipocalin (NGAL) and CRP were quantified using the Multiplex Hybcell Technology (Cube Dx GmbH, 4300 St. Valentin, Austria).

### 2.2 Blood sampling

Blood from ICU patients was drawn from 20 gauge catheters within the radial artery (8 cm polyethylene catheter, Vygon, Aachen, Germany) on the day of admission to the ICU, while patients with CAP and healthy volunteers were sampled by venipuncture using 21‐gauge needles (Safety‐Multifly, Sarstedt AG & Co, Nümbrecht, Germany). We recently showed, that arterial *vs*. venous blood sampling has insignificant effects on EV miRNA expression.^22^ Blood was drawn into 9 ml serum collection tubes (S‐Monovette, Sarstedt AG&Co, Nümbrecht, Germany) each, allowed to clot for 30 minutes and centrifuged at 3400 g for 10 minutes at room temperature (RT). Within 10 minutes of separation, serum was aliquoted and immediately stored at −80°C.

### 2.3 Sample preparation

Samples were processed according to the manufacturer's protocols. Crude EVs were precipitated (miRCURY Exosome Isolation Kit‐Serum and Plasma, Qiagen, Venlo, the Netherlands) from either 1 ml (small RNA‐seq in the training cohort and additional RT‐qPCR confirmation in the validation cohort) or 0.75 ml (technical RT‐qPCR validation in the training cohort) of serum, respectively. As previously shown by our group, in contrast to other EV isolation methods, precipitation allows for reliable separation of sepsis patients and healthy volunteers in small RNA‐seq analyses.^23^ As precipitation co‐isolates cell‐free non‐EV miRNA carriers, such as high‐ and low‐density lipoproteins, argonaute‐2 protein complexes and others,^24^, ^25^ it should be noted that miRNAs from this study are not exclusively EV‐derived. Samples were therefore designated as crude EVs. After extracting cell‐free total RNA, size distribution and yield were assessed by capillary electrophoresis using the RNA 6000 Pico Kit on the 2100 Bioanalyzer (Agilent Technologies, Santa Clara, USA). The miRNeasy Serum/Plasma Advanced Kit (Qiagen, Venlo, The Netherlands) was used for small RNA‐seq, whereas the NucleoSpin miRNA Plasma Kit (Macherey‐Nagel GmbH & Co. KG, Düren, Germany) was applied for RT‐qPCR experiments. Elution steps were performed twice to increase RNA yields. After vacuum‐induced centrifugal evaporation, RNA was diluted in 8 µl of nuclease‐free water for small RNA‐seq and 9 µl for RT‐qPCR experiments, respectively. Differences in RNA concentrations were tested with the non‐parametric Mann‐Whitney U test using Graphpad Prism (version 8.3.0) and were reported as median values and quartiles (interquartile range, IQR).

### 2.4 Small RNA sequencing

As described previously,^23^ libraries were prepared with the NEBNext Multiplex Small RNA Library Prep Set for Illumina (New England Biolabs Inc, Ipswich, USA). Six ng of each cDNA library, as well as total libraries for samples with lower concentrations were pooled. Single‐end sequencing ran in 50 cycles on the HiSeq2500 (Illumina Inc, San Diego, USA).

Quality control of small RNA‐seq data, trimming of adaptor sequences and alignment of reads was performed as described before.^22^ Only samples with a minimum of 1 million reads altogether and 15% of miRNA reads in relation to total library size were included for analyses. DGE analysis was conducted by DESeq2 (version 1.22.1) ^26^ for R (version 3.5.1) with the implemented normalisation strategy based on library size correction and the Benjamini‐Hochberg method to correct for the false discovery rate (FDR). miRNAs were filtered by setting a mean expression across all samples of ≥50 reads (baseMean), a minimum twofold up‐ or down‐regulation (log2 fold change, log2FC ≥1 or log2FC ≤ −1) and adjusted *P*‐value padj ≤ 0.05. miRNAs that failed to be detected in more than one sample per group were removed prior to selecting the most drastically dysregulated miRNAs based on log2FC. Unsupervised clustering was performed by principal component analysis (PCA). Additionally, after filtering small RNA‐seq data for baseMean ≥500, supervised clustering was performed by sparse partial‐least‐squares discriminant analysis (sPLS‐DA) with four components and maximum five features each using mixOmics^27^ to assess the minimal number of miRNAs required to separate groups. Combining data from both, DGE analysis and sPLS‐DA, a subset of candidate miRNAs serving as a whole biomarker signature was selected. Statistical significance of RNA mapping was tested with the non‐parametric Kruskal‐Wallis test followed by Dunn's multiple comparison test using Graphpad Prism (version 8.3.0). Relative mapping frequencies were reported as mean values ± standard deviation.

### 2.5 RT‐qPCR validation

The most stably expressed miRNAs among all groups were evaluated from the NGS data set as potential reference miRNAs by NormFinder.^28^ Validations of the biomarker signature were performed by RT‐qPCR using the LNA‐optimized miRNA PCR system (miRCURY LNA RT kit, miRCURY LNA SYBR Green PCR kit, Qiagen, Venlo, the Netherlands). For reverse transcription, 6.5 µl of cell‐free total RNA was used as template for cDNA synthesis. qPCR reactions were prepared according to the manufacturer's recommendation with the appropriate miRCURY LNA miRNA PCR Assays (Qiagen, Venlo, the Netherlands) for the biomarker candidates and the reference miR‐30d‐5p. The UniSp6 assay (Qiagen, Venlo, the Netherlands) was used as control for cDNA synthesis and PCR amplification. qPCR reactions were run in triplicates on a ViiA 7 Real‐Time PCR System (Thermo Fisher Scientific, Waltham, USA) with the low ROX reference dye (Qiagen, Venlo, the Netherlands). Amplification plots and melting curves were analysed using the QuantStudio Real‐Time PCR Software (version 1.3, Applied Biosystems, Waltham, USA). Assays with insufficient signal detection were excluded. Only samples with Cq (cycle quantification) values lower than 38 cycles in at least two replicates were included for analyses. Mean Cq values were quantified relatively with the ∆∆Cq method.^29^ Statistical analyses of data were performed by Graphpad Prism (version 8.3.0) using the non‐parametric Kruskal‐Wallis test followed by Dunn's multiple comparison test. miRNA expression levels from small RNA‐seq and RT‐qPCR experiments were compared by Spearman's rank‐order correlation. Spearman's r and 95% confidence intervals were reported. Additionally, group classification of RT‐qPCR data from the independent validation cohort was performed based on partial‐least‐squares discriminant analysis (PLS‐DA) of RT‐qPCR data from the training cohort. Expression values of validated miRNAs were depicted as median and IQR.

### 2.6 Statistical analysis of demographics and clinical data

Demographic characteristics and clinical data were compared using the non‐parametric Mann‐Whitney U test or in case of more than two groups by ANOVA on Ranks followed by Dunn's post hoc test. The chi‐square or Fisher's exact test was used for comparison of categorical variables. Expression levels of significantly regulated miRNAs from the NGS and the qPCR confirmation analysis were correlated with demographic and clinical data from CAP and sepsis patients of the training and validation cohorts by calculating the non‐parametric Spearman's Rank Order correlation.

Data analysis was performed using Python version 3.7 (Python Software Foundation, Beaverton, USA) and SPSS (IBM SPSS Statistics for Windows, version 25; IBM Corp., Armonk, NY, USA). Data in the text and in tables are reported as median and IQR. All statistical tests were two‐tailed, and a *P*‐value <0.05 was considered statistically significant.

### 2.7 Identification of pathways relevant to community‐acquired pneumonia

Ingenuity Pathway Analysis (IPA, Spring version 2020, Qiagen Bioinformatics, Redwood, USA) was used for the in silico identification of gene targets and causal networks from the high‐throughput miRNA expression data of the training set (n = 67 patients). Only the 29 miRNAs meeting the predefined cut‐off values (baseMean ≥50, log2FC ≥1 or log2FC ≤ −1 and padj ≤0.05) were entered into IPA, and only experimentally confirmed relationships were considered for the identification of miRNA targets and the characterisation of regulatory effects. Possible gene targets were identified using the IPA ‘*microRNA Target Filter’* to identify target genes and to construct networks of relevance to CAP. Disease filtering was set to ‘*infectious disease’*, and the network for ‘*cellular and humoral immune response’* was selected.

### 2.8 Ethics approval and consent to participate

Approval of the study was granted by the Ethics Committee of the Medical Faculty of the Ludwig‐Maximilians‐University of Munich under Protocol #551‐14. All samples were anonymised during analyses. The study was conducted in accordance with approved guidelines, and written informed consent to participate was obtained from each participant or the patient's legal representative.

## 3 RESULTS

### 3.1 Study population

Demographic data of patients with CAP were comparable to healthy volunteers with regard to Body Mass Index, but healthy individuals were significantly younger than patients with CAP (50.0, 45.5‐53.0 years vs. 73.0, 63.2‐81.0 years, *P *< 0.001). Patients with CAP and sepsis from the training and validation cohorts were comparable with no significant differences in demographic and treatment data (see Tables S2 and S3 of the supporting information). Patients with sepsis were significantly younger and had significantly higher plasma concentrations of the inflammatory markers PCT, CRP, NGAL and IL‐6 at ICU admission and a significantly longer duration of hospital stay than patients with CAP (see Table S4 of the online supplement for details).

### 3.2 RNA yield, sequencing quality and mapping distribution

As different RNA isolation kits might influence miRNA recovery and composition of RNA,^30^, ^31^ yields of crude EVs precipitated from 1 ml serum diverged with respect to individual variation and the isolation method. With 22.30 (8.18‐56.28) ng, the median amount of total RNA across all groups was significantly higher (*P* = 0.003) in the validation cohort, when compared to the 11.45 (4.67‐24.10) ng of the training cohort. The median amount of total RNA extracted from the training cohort using the miRNeasy Serum/Plasma Advanced Kit (Qiagen, Venlo, the Netherlands) was 7.26 (1.00‐11.45) ng for volunteers, 11.26 (2.73‐30.71) ng for CAP patients and 20.04 (11.94‐64.60) ng for patients with sepsis. With the NucleoSpin miRNA Plasma Kit (Macherey‐Nagel GmbH & Co. KG, Düren, Germany) total RNA yields from the validation cohort were 8.13 (5.83‐16.38) ng for volunteers, 21.97 (8.79‐54.27) ng for patients with CAP and 45.66 (27.16‐95.22) ng for sepsis patients.

FastQC^32^ evaluated high per‐base sequence quality (base 1‐30) for all samples with median Phred scores of either 38 or 40. Across all samples, 1894 different miRNAs were detected with at least one read in one sample. Out of these, 225 miRNAs showed an expression level of baseMean ≥50 reads, corresponding to the filter, that was set as the expression minimum during DGE analysis.

**FIGURE 1:**
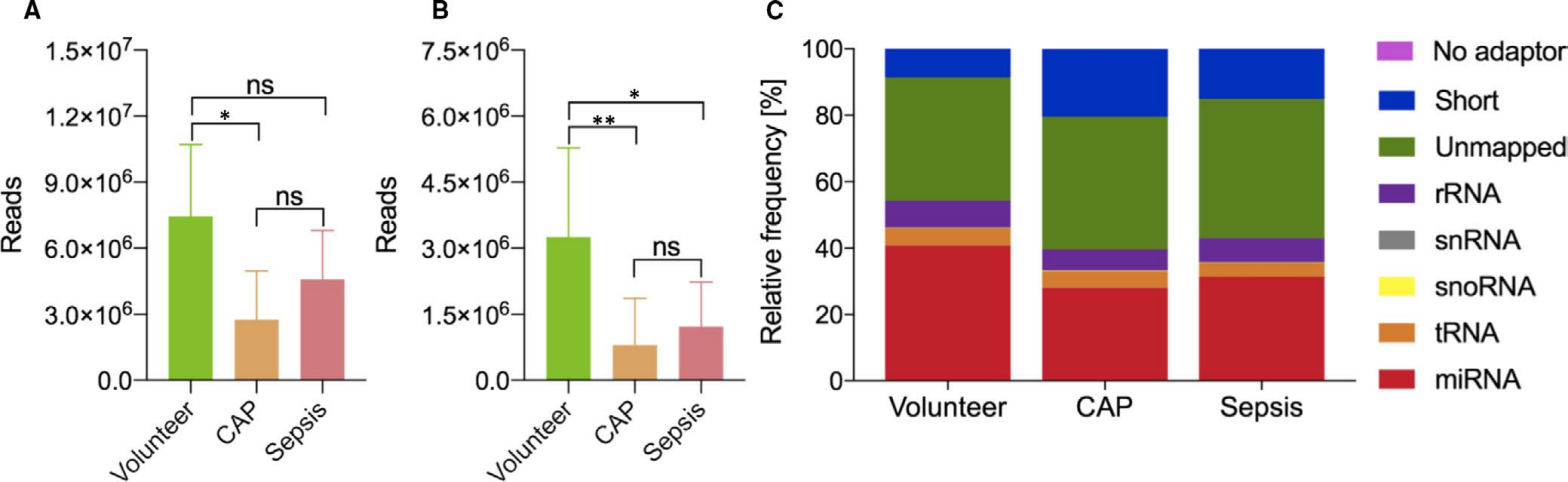
Library sizes (A) and mapped miRNA reads (B) are depicted for volunteers, patients with community‐acquired pneumonia (CAP) and sepsis as median values and 95% confidence intervals. Library sizes, as well as numbers of mapped miRNA reads tended to be higher in volunteers compared to patients, with no apparent difference between patient groups. Read counts in the mapping distribution (C) are visualised as mean relative frequencies. Volunteers have higher relative miRNA frequencies, as well as fewer incidences of short sequences (<16 nucleotides) when compared to patient groups. No adaptor: sequence lacking adaptors; short: sequence < 16 nucleotides; unmapped: sequence did not align to any of the mapped RNA classes; rRNA: ribosomal RNA; snoRNA: small nucleolar RNA; snRNA: small nuclear RNA; tRNA: transfer RNA; miRNA: microRNA; * P < 0.05; ** P < 0.005; ns: not significant

Library sizes (Figure 1A), as well as numbers of mapped miRNAs (Figure 1B) tended to be higher in volunteers when compared to patients, while no apparent difference was present between both patient groups. Relative miRNA frequencies were 40.9 ± 11.3% for volunteers, 28.0 ± 9.3% for CAP patients and 31.5 ± 14.4% for patients with sepsis. Additionally, patient groups had higher frequencies of short sequences < 16 nucleotides in size (CAP: 20.3 ± 15.6%, sepsis: 15.0 ± 12.3%) when compared to volunteers 8.5 ± 8.4%, which probably represent degradation products from longer coding and non‐coding RNA species (CAP vs. volunteers: *P* = 0.012, sepsis *vs*. volunteers: *P* = 0.061, CAP vs. sepsis: *P* = 0.831). The mapping distribution is visualised by relative mean frequencies (Figure 1C).

### 3.3 Small RNA sequencing data analyses

**FIGURE 2:**
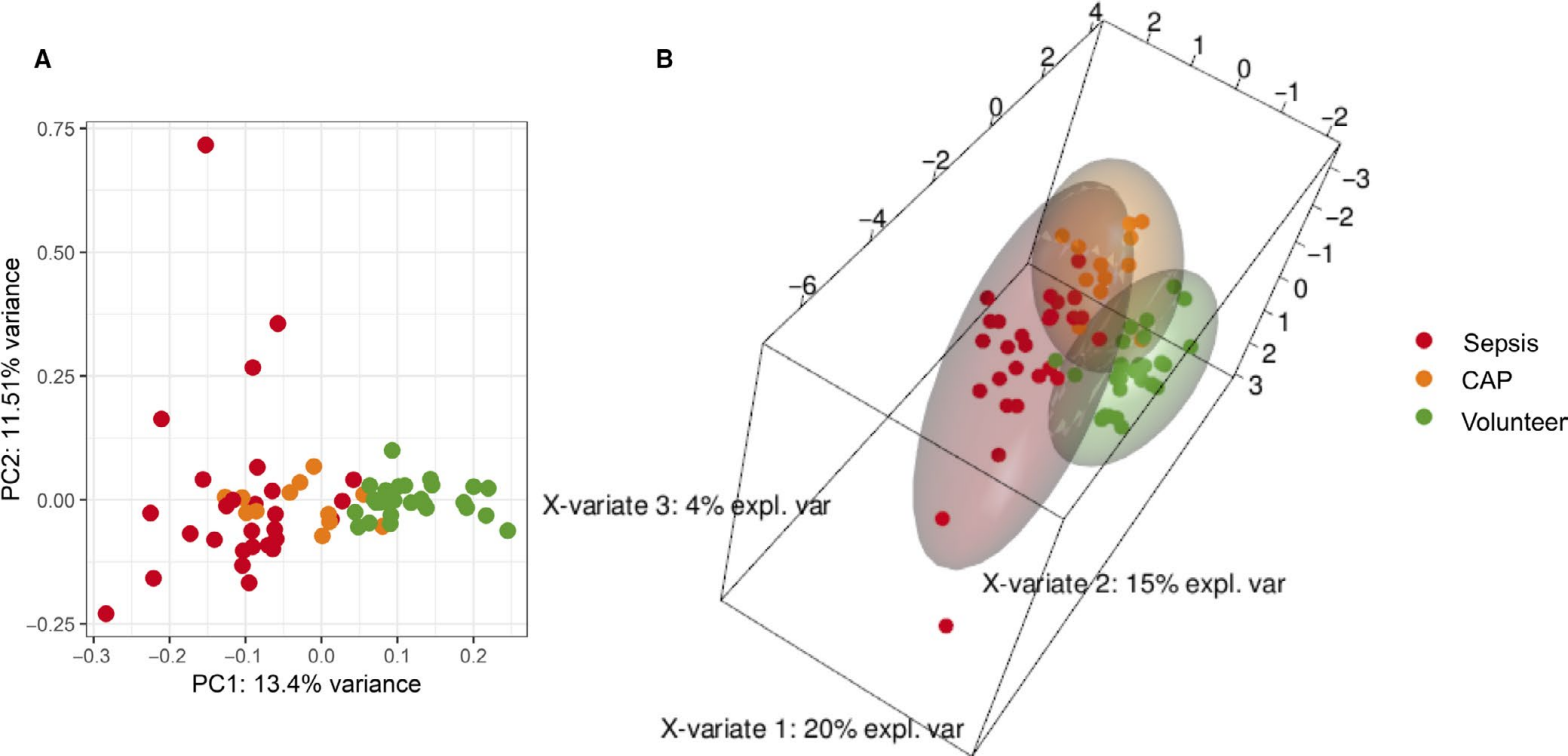
Multivariate analyses of small RNA‐sequencing data. Unsupervised clustering (A) by principal component analysis based on the 500 miRNAs with highest variance. Volunteers clustered separately from patient groups. Sepsis patients were separated more distinctly from volunteers than patients with community‐acquired pneumonia (CAP). Sparse partial‐least‐squares discriminant analysis for components 1‐3 (B) discriminated different groups by twelve miRNAs. PC: principal component; expl. var: explained variance

When performing unsupervised clustering based on the 500 miRNAs with highest variance, different patient groups overlapped, but could be distinctly separated from volunteers (Figure 2A). Patients with CAP and lower overall disease severity tended to reflect the miRNA profiles of volunteers more closely than sepsis patients, who were more distinctly separated.

Analysing DGE data, the comparison of CAP patients with volunteers revealed 29 significantly up‐ or down‐regulated miRNAs (see Table S5 of the supporting information).

Among these cell‐free miRNAs, expression levels of several transcripts correlated with indicators of disease severity in CAP patients including the CURB‐65 score as assessment score for pneumonia and the associated risk of mortality,^8^ total duration of hospital stay, and plasma levels of NGAL (see Table 1 for details). Only miR‐127‐3p was related to demographic variables of CAP patients (age: *r* = 0.627, *P* = 0.029) but none of the other miRNAs appeared to be influenced by demographics.

**TABLE 1:** Correlational matrix showing the relationship between demographic and treatment variables and expression values of significantly regulated miRNAs from the NGS training set comparing patients with community‐acquired pneumonia and healthy volunteers. Data are Spearman's correlational coefficients/P‐value. miRNAs were derived from Table S5 presented in the online supplement

miRNA	Age	CURB‐65^a^ Score	Total hospital stay	Leucocyte count	NGAL^b^
miR‐127‐3p	0.627/0.029	0.587/0.045	‐	‐	−0.664/0.018
miR‐193b‐5p	‐	0.667/0.018	‐	‐	−0.832/0.001
miR‐193a‐5p	‐	‐	0.789/0.002	‐	‐
miR‐215‐5p	‐	−0.613/0.034	‐	‐	‐
miR‐450b‐5p	‐	‐	‐	0.636/0.035	‐
miR‐452‐5p	‐	‐	‐	−0.800/0.003	‐
miR‐320d	‐	‐	‐	‐	−0.636/0.026
miR‐338‐5p	‐	‐	‐	‐	0.699/0.011
miR‐379‐5p	‐	‐	‐	‐	−0.860/<0.001

^a^ Confusion, Urea, Respiratory Rate, Blood Pressure and Age score for pneumonia severity.

^b^ Neutrophil gelatinase‐associated lipocalin.

Comparing patients with CAP to those with sepsis, we detected 25 miRNAs with altered expression (see Table S5 of the supporting information). A number of these miRNAs correlated significantly with treatment variables, indicating face validity of these transcripts. In particular, expression values of miR‐1‐3p were related to plasma concentrations of the inflammatory markers IL‐6, NGAL and vasopressor requirements in sepsis (see Table 2 for details).

**TABLE 2:** Correlational matrix showing the relationship between demographic, treatment variables, inflammatory markers (lower part of the table) and expression values of significantly regulated miRNAs from the NGS training set comparing patients with community‐acquired pneumonia and those with sepsis. Data are Spearman's correlational coefficients/*P*‐value. miRNAs were derived from Table S5 presented in the online supplement

miRNA	Age	APACHE II‐Score	Duration of ICU‐treatment	Total hospital stay	Duration of mechanical ventilation
miR‐1246	−0.529/0.004	‐	‐	0.418/0.042	‐
miR‐1‐3p	−0.447/0.017	‐	‐	‐	‐
miR‐18a‐3p	0.436/0.013	‐	−0.411/0.046	−0.433/0.024	−0.400/0.035
miR‐150‐3p	‐	0.390/0.044	0.475/0.019	‐	‐
miR‐92a‐3p	‐	‐	−0.473/0.019	‐	‐
miR‐93‐5p	‐	‐	−0.496/0.014	−0.413/0.045	‐
miR‐511‐5p	‐	‐	‐	0.425/0.038	‐

miRNA	PCT^a^	CRP^b^	IL‐6^c^	NGAL^d^	Vasopressor requirement^e^
miR‐150‐3p	−0.483/0.009	‐	‐	‐	‐
miR‐4433b‐3p	−0.452/0.016	‐	‐	‐	‐
miR‐1228‐5p	‐	−0.494/0.009	‐	‐	‐
miR‐1‐3p	‐	‐	0.460/0.014	0.423/0.028	0.577/0.002
miR‐500a‐3p	‐	‐	‐	‐	0.458/0.016
miR‐95‐3p	‐	‐	‐	‐	0.493/0.009
miR‐660‐5p	‐	‐	‐	‐	0.469/0.014

^a^ Procalcitonin.

^b^ C‐reactive protein.

^c^ Interleukin‐6.

^d^ Neutrophil gelatinase‐associated lipocalin.

^e^ Vasopressor requirements are represented by the required dosage of norepinephrine to achieve an adequate mean arterial pressure to maintain organ perfusion in sepsis.

In order to establish an integrated biomarker signature, the three most drastically dysregulated miRNAs were selected from each DESeq2 comparison (CAP vs. volunteers: miR‐582‐3p, miR‐432‐5p, miR‐542‐3p; CAP *vs*. sepsis: miR‐1246, miR‐1‐3p, miR‐4433b‐3p).

Based on sPLS‐DA, group separation was achieved on the basis of twelve miRNAs as discriminators (Figure 2B). Out of these, four miRNAs (miR‐182‐5p, miR‐193a‐5p, miR‐215‐5p, miR‐93‐5p) were also detected using DESeq2 with log2FC ≥1 or log2FC ≤ −1 (see Table S5 of the supporting information for comparison), while all other miRNAs showed no regulation according to our threshold settings for DGE analysis.

Combining the respective miRNAs from both multivariate analyses, a subset of eighteen miRNAs (miR‐432‐5p, miR‐542‐3p, miR‐582‐3p, miR‐1246, miR‐1‐3p, miR‐4433b‐3p, miR‐181b‐5p, miR‐182‐5p, miR‐186‐5p, miR‐193a‐5p, miR‐199a‐3p = miR‐199b‐3p, miR‐21‐5p, miR‐215‐5p, miR‐30e‐5p, miR‐340‐5p, miR‐425‐5p, miR‐93‐5p, miR‐941) was identified as potential biomarker signature.

### 3.4 RT‐qPCR validation

The eighteen candidate miRNAs were quantified by RT‐qPCR, as a supplementary method, in the same cohort that was used for high‐throughput sequencing and additionally confirmed in an independent second cohort of individuals. After quality control of RT‐qPCR data, twelve miRNAs remained for analyses.

**FIGURE 3:**
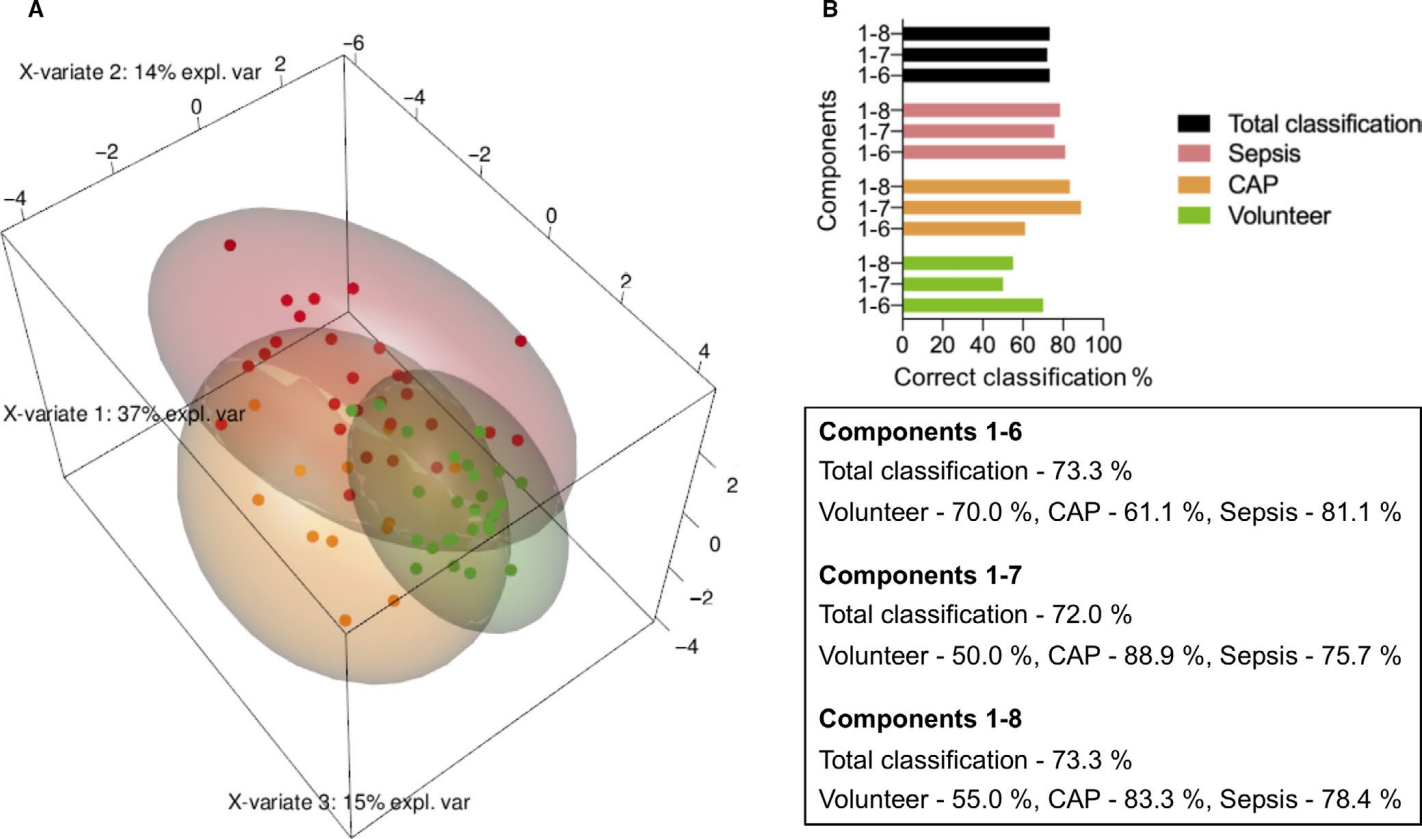
Multivariate analyses of reverse transcription quantitative real‐time PCR (RT‐qPCR) data. Partial‐least‐squares discriminant analysis for components 1‐3 (A). Based on expression levels (ΔCq) of the twelve miRNAs from the RT‐qPCR validation in the training cohort, groups were separated. Correct total classification of the validation cohort based on expression (ΔCq) of the twelve miRNAs from the training cohort and assignment for each individual group (B). expl. var: explained variance

By reading in RT‐qPCR data of all twelve miRNAs measured in the training cohort to PLS‐DA, groups were separated and equally distributed compared to the small RNA‐seq data (Figure 3A). According to findings from PLS‐DA, samples in the validation cohort could be correctly classified using principal components 1‐10 and displayed high discriminatory power with 73.3% of correctly assigned samples for reduced models from principal components 1‐6 or 1‐8, respectively (Figure 3B).

**FIGURE 4:**
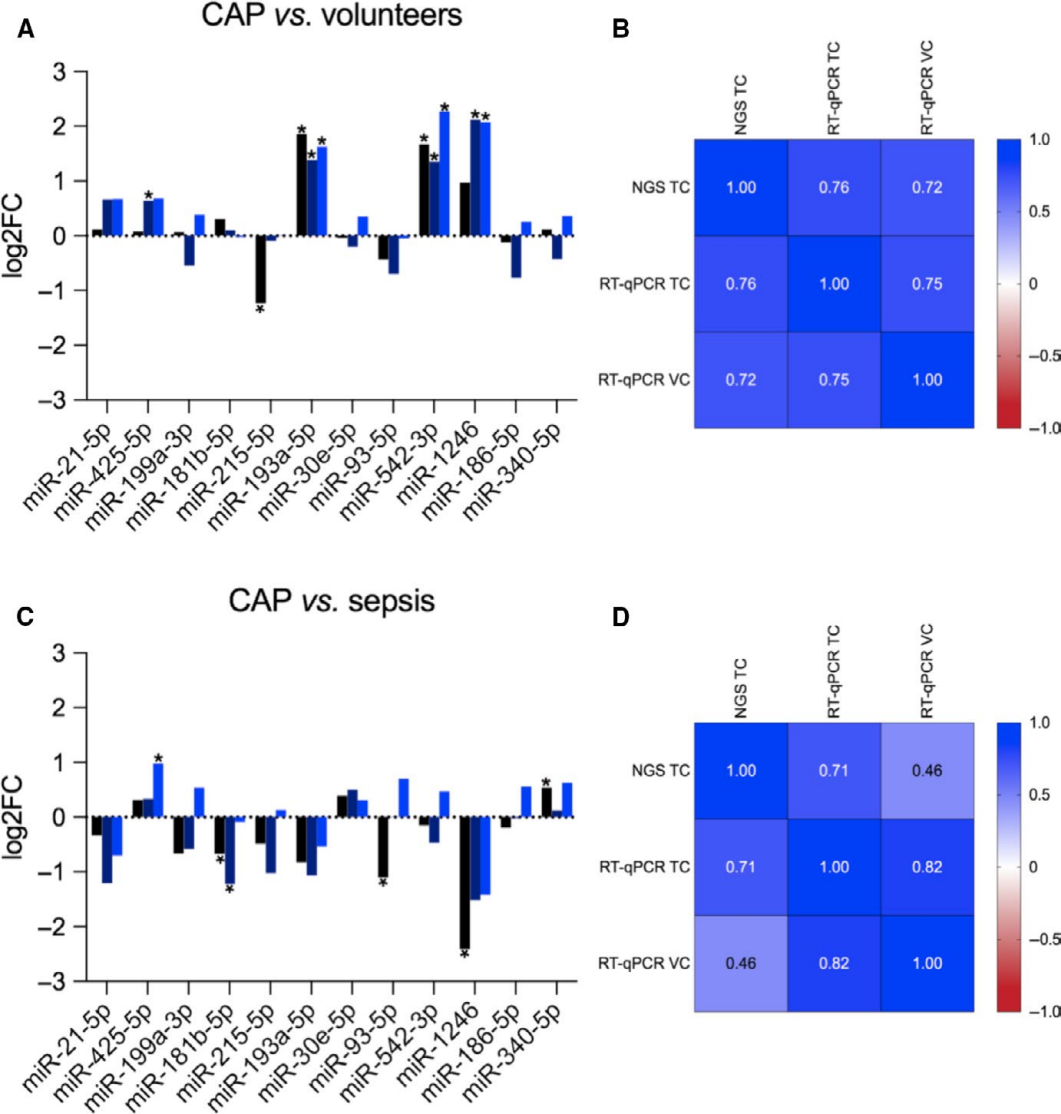
Log2 fold changes (log2FC) for CAP vs. volunteers (a), and CAP vs. sepsis (c) calculated from next‐generation sequencing (NGS, black) and reverse transcription quantitative real‐time PCR (RT‐qPCR) data in the training (TC, dark blue) and validation cohort (VC, light blue). Significant changes are marked by asterisks. Spearman's correlation matrix of expression levels (log2FC) from NGS and RT‐qPCR data sets shown as heatmaps for CAP vs. volunteers (b) and CAP vs. sepsis (d). CAP vs. volunteers: NGS TC vs. RT‐qPCR TC, *r* = 0.76 (0.32‐0.93), *P* = 0.006; NGS TC vs. RT‐qPCR VC, *r* = 0.72 (0.23‐0.92), *P* = 0.011; RT‐qPCR TC vs. RT‐qPCR VC: *r* = 0.75 (0.29‐0.93), *P* = 0.007; CAP vs. sepsis: NGS TC vs. RT‐qPCR TC, *r* = 0.71 (0.22‐0.92), *P* = 0.012; NGS TC vs. RT‐qPCR VC, *r* = 0.46 (−0.17 − 0.82), *P* = 0.13; RT‐qPCR TC vs. RT‐qPCR VC: *r* = 0.82 (0.45‐0.95), *P* = 0.002; CAP: community‐acquired pneumonia

Out of these miRNAs, some transcripts showed the same expression pattern for both group comparisons independent of cohort and approach used for analysis, albeit with reversed trends (Figure 4A and C). With one exception, Spearman's rank‐order correlation of miRNA expression from the NGS and RT‐qPCR data sets showed an overall significant positive relationship (Figure 4B and D). The three most drastically dysregulated miRNAs (CAP *vs*. volunteers: miR‐193a‐5p, miR‐542‐3p and miR‐1246; CAP *vs*. sepsis: miR‐1246) with equal expression pattern for both approaches and cohorts of individuals were selected as candidate biomarkers for further analysis.

### 3.5 Expression levels of candidate miRNAs according to subgroups

**FIGURE 5:**
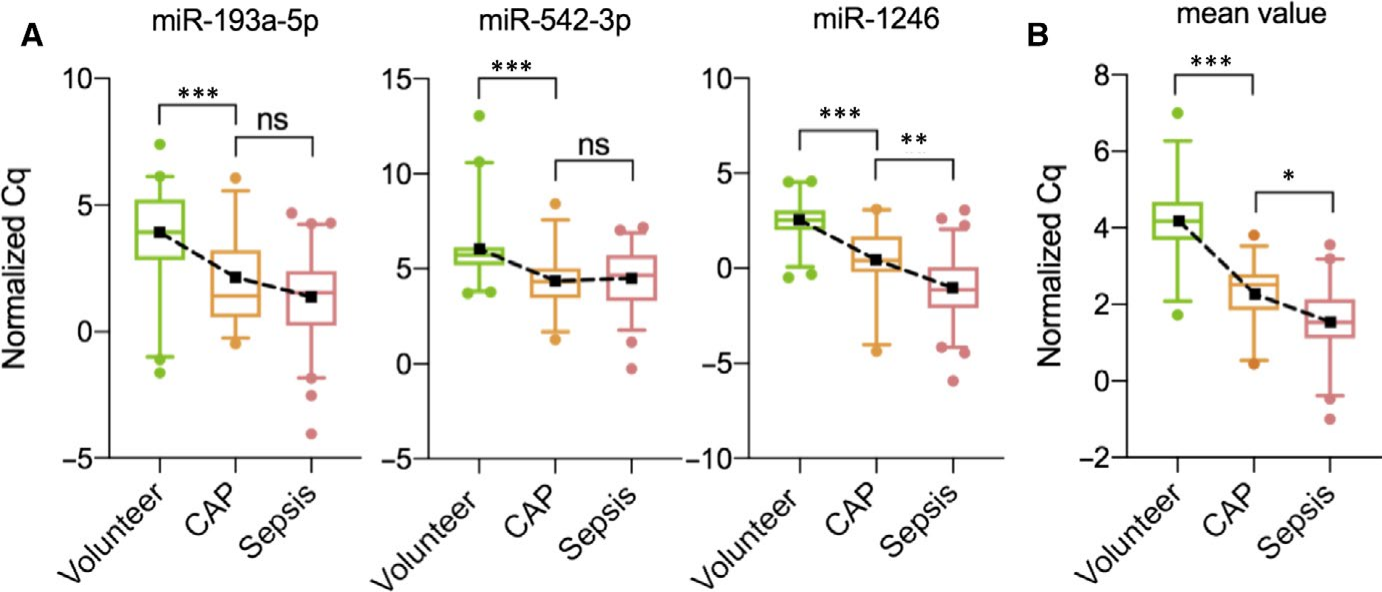
Merge of technically and additionally validated miRNAs from reverse transcription quantitative real‐time PCR experiments for each miRNA separately (A), and mean values of all three miRNAs (B). Data are displayed as boxplots from the 5th to the 95th percentile, showing median (line) and mean (square) values. Dots represent samples outside the percentile range. Changes in mean miRNA expression among groups are indicated by dashed lines. Lower values of ΔCq indicate higher expression levels of the respective miRNAs. Cq: cycle quantification; *P < 0.05; **P = 0.005; ***P < 0.001; ns: not significant

When analysing miRNA expression levels in all individuals from both the training and validation cohort together (Figure 5), normalised ΔCq values of miR‐193a‐5p in patients with CAP were significantly lower (ΔCq = 1.41, 0.57‐3.21) than in healthy volunteers (ΔCq = 3.93, 2.83‐5.22, *P *< 0.001) with no significant difference to patients with sepsis (ΔCq = 1.53, 0.24‐2.39). For miR‐542‐3p, a similar pattern was observed. Normalised ΔCq values were again significantly lower in patients with CAP (ΔCq = 4.33, 3.46‐5.02) than in healthy individuals (ΔCq = 5.72, 5.19‐6.13, *P *< 0.001) with no significant difference to sepsis (ΔCq = 4.66, 3.31‐5.71). Normalised Cq values of miR‐1246 showed a significant decrease from healthy individuals (ΔCq = 2.54, 2.00‐3.05) to patients with CAP (ΔCq = 0.41, −0.22‐1.68, *P *< 0.001) to those with sepsis (ΔCq=−1.14, −2.10‐0.07, *P* = 0.005). Overall, these findings indicate higher expression values of the respective miRNAs with higher disease severity.

### 3.6 ‘The cellular and humoral immune response’ in community‐acquired pneumonia

When IPA target filtering was set to ‘*cellular and humoral immune response’*, the resulting canonical network in CAP patients was primarily affected by 6 of the 29 significantly regulated miRNAs with 42 possible target mRNA transcripts. Figure S1 in the online supplement shows a simplified network of the two validated and up‐regulated miRNAs miR‐193a‐5p and miR‐542‐3p, their possible mRNA targets and their interaction. Possible molecular targets identified by applying the IPA ‘*microRNA Target Filter’* module for the candidate miRNA miR‐193a‐5p were IL‐10 (Interleukin‐10), IL2RG (Interleukin‐2 receptor subunit gamma) and mTOR (mechanistic Target of Rapamycin). One of the identified molecular targets of miR‐542‐3p was inflammatory mediator PTGS2 (prostaglandin G/H synthase 2).

## 4 DISCUSSION

Our study outlines the possibility of using cell‐free miRNA biomarkers to discriminate patients with CAP from healthy volunteers and from those with sepsis as a severe secondary complication. We characterised a subset of twelve miRNAs as a potential biomarker for this purpose, technically validated miR‐193a‐5p, miR‐542‐3p and miR‐1246 and confirmed our findings in an independent cohort.

When analysing relative miRNA expression, miR‐1246 showed significant increases with more severe overall disease from volunteers to patients with CAP and to those with sepsis, whereas miR‐193a‐5p and miR‐542‐3p differentiated patients with an infectious disease (CAP or sepsis) from healthy individuals.

The miR‐193a/b‐5p family was previously shown to be a possible indicator for CAP, as expression of miR‐193b‐5p was related to the CURB‐65 score, a validated clinical prediction score for pneumonia and the associated risk of mortality^8^ and also to NGAL plasma levels. NGAL has recently been described as a useful biomarker for lower respiratory tract infections^33^ and the associated mortality^34^ and may also serve as an indicator of an ongoing risk for renal injury,^35^ which is common in patients with pulmonary disorders.^36^ Moreover, expression levels of miR‐193a‐5p were related to the duration of the required hospital stay in our study. Identified molecular targets of miR‐193a‐5p included IL‐10, which is known to correlate with the CURB‐65 score and is associated with increased mortality in patients with CAP^37^ and mTOR, which has been shown to be down‐regulated in sepsis due to CAP.^38^ Our group previously showed the positive correlation of cell‐free miR‐193a‐5p expression with disease severity in sepsis patients.^17^ Expression levels of miR‐193 in serum were also associated with death from sepsis in recent studies.^39^ miR‐542‐3p was shown to be a causal mediator of mitochondrial dysfunction in muscle tissue of patients with sepsis.^40^ In our study, one of the molecular targets of miR‐542‐3p was PTGS2, a pro‐inflammatory mediator, which is known to be responsible for the production of prostaglandins that are involved in the inflammatory response.^41^ Moreover, elevated miR‐1246 levels have been shown to mediate lipopolysaccharide‐induced apoptosis of pulmonary endothelial cells and acute lung injury.^42^ In conclusion, our findings indicate that these three cell‐free miRNAs may be valuable for the diagnosis of CAP and sepsis as a severe secondary complication.

The fact that these miRNAs were also associated to EVs isolated from the peripheral circulation suggests a possible role of these vesicles in mediating inflammatory signals from the lung to the periphery including blood cells or *vice versa* by acting as a transport media for non‐coding RNAs and other signalling molecules. A recent study in animals with experimental bacterial pulmonary infection clearly demonstrated the existence of a subpopulation of EVs in bronchoalveolar lavage fluid that contained high concentrations of pro‐inflammatory miRNAs and was likely derived from alveolar epithelial type‐I cells.^43^ These EVs could theoretically reach the peripheral circulation, but the responsible mechanisms for transport of EVs within the lung and to the periphery are poorly understood.^44^ Our observation that the expression values of some of the differentially expressed miRNAs isolated from crude EVs clearly correlated with clinical indicators of pneumonia severity points to this possibility but does not prove it.

A limitation of our study results from the fact that two different RNA extraction methods were used for small RNA‐seq and RT‐qPCR. As miRNA composition might vary with the respective RNA isolation method,^30^, ^31^ and different approaches were used, our data might be biased partially. Given the fact that individual biological properties and different miRNA analysing platforms (NGS and RT‐qPCR) are associated with miRNA expression profiles,^45^, ^46^ this might have contributed to reversed trends in miRNA regulation seen with some of the miRNAs identified in our study. However, the fact that our final biomarker signature has been confirmed for two different RNA extraction methods (1), miRNA analysing platforms (2) and individual cohorts (3) might represent additional proof of its stability.

In conclusion, our findings indicate that in patients with suspected CAP, cell‐free miR‐193a‐5p, miR‐miR‐542‐3p and miR‐1246 may serve as indicators for CAP, whereas a further increase in miR‐1246 may suggest an increased risk to develop sepsis. For the successful use of these miRNAs as biomarkers, studies in larger patient cohorts with both conditions will be required to confirm our data of this novel diagnostic approach.

## CONFLICT OF INTEREST

The authors confirm that there are no conflicts of interest.

## AUTHORS’ CONTRIBUTION

Stefanie Hermann: Conceptualization (supporting); Data curation (lead); Formal analysis (lead); Visualization (lead); Writing‐original draft (lead). Florian Brandes: Conceptualization (supporting); Data curation (lead); Formal analysis (lead); Writing‐original draft (lead). Benedikt Kirchner: Conceptualization (supporting); Formal analysis (supporting); Visualization (supporting); Writing‐review & editing (equal). Dominik Buschmann: Conceptualization (supporting); Formal analysis (supporting); Writing‐review & editing (equal). Melanie Borrmann: Conceptualization (supporting); Formal analysis (supporting); Writing‐review & editing (equal). Matthias Klein: Conceptualization (supporting); Writing‐review & editing (equal). Stefan Kotschote: Conceptualization (supporting); Funding acquisition (lead); Writing‐review & editing (equal). Michael Bonin: Conceptualization (supporting); Funding acquisition (lead); Writing‐review & editing (equal). Marlene Reithmair: Conceptualization (lead); Funding acquisition (lead); Project administration (lead); Writing‐review & editing (equal). Ines Kaufmann: Conceptualization (supporting); Writing‐review & editing (equal). Gustav Schelling: Conceptualization (lead); Data curation (lead); Formal analysis (lead); Writing‐original draft (lead). Michael W Pfaffl: Conceptualization (lead); Funding acquisition (lead); Project administration (lead); Writing‐review & editing (equal).

## Supporting information

Supplementary Material

## Data Availability

Small RNA‐seq data were deposited with the European Nucleotide Archive (http://www.ebi.ac.uk/ena/data/view/PRJEB35757). RT‐qPCR data are available from the corresponding author upon reasonable request.
