# Supplementary material for: Diagnostic potential of circulating cell‐free microRNAs for community‐acquired pneumonia and pneumonia‐related sepsis
Source: J Cell Mol Med. 2020 Sep 11;24(20):12054–64. doi: 10.1111/jcmm.15837 (PMC7578906; doi:10.1111/jcmm.15837)
Supplement: Supplementary file 1 — Supplementary Material [file JCMM-24-12054-s001.docx]

**Diagnostic potential of circulating cell-free microRNAs for community-acquired pneumonia and pneumonia-related sepsis**

Stefanie Hermann^1†‡^, Florian Brandes^2†^, Benedikt Kirchner^1^, Dominik Buschmann^1^, Melanie Borrmann^2^, Matthias Klein^3^, Stefan Kotschote^4^, Michael Bonin^4^, Marlene Reithmair^5^, Ines Kaufmann^6^, Gustav Schelling^2^, Michael W. Pfaffl^1^

^1^Division of Animal Physiology and Immunology, School of Life Sciences Weihenstephan, Technical University of Munich, Freising, Germany

^2^Department of Anesthesiology, University Hospital, Ludwig-Maximilians-University Munich, Munich, Germany

^3^Department of Neurology, University Hospital, Ludwig-Maximilians-University of Munich, Munich, Germany

^4^IMGM Laboratories GmbH, Planegg, Germany

^5^Department of Anesthesia, Klinikum Neuperlach, Munich City Hospitals, Munich, Munich, Germany

^5^Institute of Human Genetics, University Hospital, Ludwig-Maximilians-University Munich, Munich, Germany

^6^Department of Anesthesia, Klinikum Neuperlach, Munich City Hospitals, Munich, Germany

^†^ SH and FB should be considered joint first author.

^‡^ Corresponding author

***Supporting information***

**Table S1**: Study inclusion and exclusion criteria according to groups.

| **Criteria** | **Sepsis** | **CAP**^‡^ | **Volunteer** |
| --- | --- | --- | --- |
| **Inclusion** | Sepsis or septic shock according to Sepsis-3 criteria [1] | Patients with community-acquired pneumonia without recent hospitalization or association with other healthcare facilities such as nursing homes, dialysis centers, and outpatient clinics.  CURB-65^†^ ≥ 1 and clinical symptoms like fever, cough and dyspnea. | Charlson Comorbidity Index = 0 [2] |
| **Exclusion** | No consent given by patients or next-of-kin | | |
|  | Age < 18 | | |
|  | Pregnancy | | |
|  | Preexisting chronic infectious disorders (e.g. endocarditis, HIV or hepatitis) | | |
|  | Current tumor or malignant disorders | | |
|  | Limited patient’s life expectancy < 6 months (independent of e.g. sepsis/SIRS, pneumonia or localized infection) | | |
|  | Immunosuppression or steroid therapy (autoimmune disease, transplantation) | | |

^†^Confusion, Urea, Respiratory Rate, Blood Pressure and Age (CURB-65) score for pneumonia severity [3]

^‡^CAP: community-acquired pneumonia

**Table S2**: Comparison of demographic and clinical data between patients with community-acquired pneumonia from the training and the validation cohort. Measurements were taken at study inclusion in the emergency room.

| **Parameter** | **Training cohort (n=12)** | **Validation cohort (n=18)** | **p-value** |
| --- | --- | --- | --- |
| Age (years) | 72.0 (64.8-85.2) | 73.5 (60.5-78.8) | 0.372 |
| Body Mass Index (kg/m^2^) | 27.5 (24.1-30.3) | 24.9 (22.2-30.6) | 0.187 |
| Duration of hospital therapy (days) | 10.5 (8.5-14.2) | 5.0 (4.5-6.0) | 0.056 |
| Serum creatinine (mg/dl) | 1.2 (1.0-1.6) | 1.4 (1.2-1.6) | 0.227 |
| Serum urea nitrogen (mg/dl) | 40.0 (27.0-74.8) | 57.0 (50.0-76.0) | 0.170 |
| CURB-65 score^†^ | 1.0 (1.0-2.0) | 1.0 (1.0-2.0) | 0.285 |
| Serum procalcitonin (ng/ml) | 0.2 (0.1-0.6) | 0.3 (0.2-2.7) | 0.162 |
| Leucocyte count (G/l) | 9.0 (6.8-12.5) | 8.7 (7.6-14.0) | 0.451 |
| C-reactive protein (ug/dl) | 11.8 (2.4-19.0) | 16.8 (5.8-27.3) | 0.132 |

Data are median and interquartile range.

^†^Confusion, Urea, Respiratory Rate, Blood Pressure and Age (CURB-65) score for pneumonia severity [3]

**Table S3**: Comparison of demographic and clinical data between patients with sepsis from the training and the validation cohort. Measurments were taken at study inclusion (admittance to the intensive care unit, ICU).

| **Parameter** | **Training cohort (n=28)** | **Validation cohort (n=37)** | **p-value** |
| --- | --- | --- | --- |
| Age (years) | 66.5 (60.2-75.0) | 68.0 (54.2-73.8) | 0.406 |
| Body Mass Index (kg/m^2^) | 23.8 (21.8-28.9) | 27.4 (23.4-30.4) | 0.098 |
| SAPS Score^†^ | 47.5 (26.0-60.5) | 63.5 (53.2-77.2) | 0.004 |
| APACHE II Score^‡^ | 23.0 (15.5-29.5) | 32.0 (23.8-36.0) | 0.015 |
| SOFA Score^§^ | 11.5 (9.0-12.8) | 14.0 (12.0-18.0) | 0.001 |
| Duration of ICU therapy (days) | 12.0 (9.0-32.5) | 8.5 (5.5-21.2) | 0.084 |
| Duration of mechanical ventilation (days) | 10.5 (6.5-24.2) | 13.0 (2.0-21.5) | 0.256 |
| Serum lactate (mmol/l) | 2.7 (1.6 - 4.0) | 2.6 (2.2-3.6) | 0.384 |
| Noradrenalin dosage (µg/kg/min) | 0.3 (0.1-0.6) | 0.4 (0.1-0.8) | 0.243 |
| Serum creatinine (mg/dl) | 1.4 (0.8-2.0) | 1.6 (0.8-2.4) | 0.258 |
| Serum urea nitrogen (mg/dl) | 52.5 (38.0-79.5) | 62.0 (46.0-101.0) | 0.186 |
| Neutrophil gelatinase-associated lipocalin (NGAL) (ng/ml) [4] | 125.0 (36.0-273.0) | 152.0 (112.8-183.5) | 0.488 |
| Cystatin C (ng/ml) [5] | 1.7 (1.0-2.9) | 0.8 (0.8-1.2) | 0.055 |
| Procalcitonin (ng/ml) | 4.7 (2.2-9.4) | 4.2 (1.6-12.3) | 0.463 |
| Leucocyte count (G/l) | 14.6 (8.2-18.5) | 14.7 (11.4-20.1) | 0.296 |
| C-reactive protein (ug/dl) | 22.0 (15.7-31.1) | 20.7 (11.5-29.4) | 0.155 |
| Interleukin-6 (pg/ml) | 236.0 (97.0-1762.0) | 278.0 (48.0-1442.0) | 0.464 |

Data are median and interquartile range or number per group. ^†^Simplified Acute Physiology Score II [6]

^‡^Acute Physiology And Chronic Health Evaluation score [7]

^§^Sepsis-related Organ Failure Asssessment Score [8]

**Table S4**: Comparison of demographic and clinical data between patients with community-acquired pneumonia (CAP) and patients with sepsis (training and validation cohorts combined). Measurements were performed at hospital (CAP) and intensive care unit admission (sepsis), respectively.

| **Parameter** | **CAP (n=30)** | **Sepsis (n=65)** | **p-value** |
| --- | --- | --- | --- |
| Age (years) | 73.0 (63.2-81.0) | 67.0 (55.8-75.0) | 0.019 |
| Body Mass Index (kg/m^2^) | 25.2 (23.1-30.5) | 25.7 (22.0-29.3) | 0.307 |
| Duration of hospital therapy (days) | 10.0 (6.0-12.5) | 26.0 (14.0-38.5) | <0.001 |
| Sepsis^†^ (Sepsis/Septic shock) | - | 11/54 | - |
| Serum creatinine (mg/dl) | 1.2 (1.1-1.6) | 1.4 (0.8-2.1) | 0.37 |
| Serum urea nitrogen (mg/dl) | 50.0 (32.0-76.0) | 57.0 (41.0-89.0) | 0.210 |
| Neutrophil gelatinase-associated lipocalin (NGAL) (ng/ml) [4] | 255.0 (165.2-628.8) | 135.0 (36.0-262.0) | 0.015 |
| Cystatin C (ng/ml) [5] | 1.7 (1.6-2.0) | 1.6 (0.9-2.7) | 0.228 |
| Procalcitonin (ng/ml) | 0.2 (0.1-0.7) | 4.7 (1.8-9.8) | <0.001 |
| Leucocyte count (G/l) | 9.0 (7.0-13.2) | 14.7 (10.2-19.1) | 0.002 |
| C-reactive protein (ug/dl) | 11.8 (4.2-24.5) | 21.4 (14.7-30.4) | 0.010 |
| Interleukin-6 (pg/ml) | 13.0 (0.0-26.6) | 255.0 (69.0-1580.5) | <0.001 |

Data are median and interquartile range or number per group.

^†^Sepsis was defined according to Sepsis-3 criteria [1].

**Table S5**: DESeq2 regulated miRNAs for the comparison of patients with community-acquired pneumonia (CAP) to volunteers and to sepsis patients. microRNAs (miRNAs) are sorted in descending order by regulation (log2FC).

| **CAP *vs.* Volunteers** | | | | **CAP *vs.* Sepsis** | | | |
| --- | --- | --- | --- | --- | --- | --- | --- |
| **miRNA** | **bMean**^†^ | **log2FC**^‡^ | **padj**^§^ | **miRNA** | **bMean**^†^ | **log2FC**^‡^ | **padj**^§^ |
| miR-582-3p | 585.82 | 2.53 | 1.81E-09 | miR-1228-5p | 79.86 | 4.70 | 1.08E-04 |
| miR-193a-5p | 1237.19 | 1.86 | 1.49E-06 | miR-4433b-3p | 402.55 | 3.36 | 1.67E-05 |
| miR-542-3p | 63.20 | 1.67 | 3.29E-05 | miR-4446-3p | 107.61 | 2.05 | 3.93E-04 |
| miR-885-5p | 61.32 | 1.64 | 3.49E-02 | miR-370-3p | 327.22 | 1.28 | 1.18E-02 |
| miR-193b-5p | 81.48 | 1.50 | 1.22E-02 | let-7e-5p | 238.28 | 1.26 | 4.42E-03 |
| miR-452-5p | 57.00 | 1.37 | 7.59E-03 | miR-11400 | 189.74 | 1.08 | 8.66E-03 |
| miR-450b-5p | 75.95 | 1.31 | 1.37E-03 | miR-150-3p | 70.16 | 1.04 | 2.67E-02 |
| miR-27a-5p | 82.11 | 1.29 | 2.83E-03 | miR-378a-3p | 2574.52 | -1.03 | 4.05E-03 |
| miR-338-5p | 212.18 | 1.28 | 1.67E-04 | miR-378c | 67.89 | -1.09 | 8.56E-03 |
| miR-148a-3p | 304143.06 | 1.18 | 3.37E-03 | miR-93-5p | 620.40 | -1.10 | 3.09E-03 |
| miR-223-5p | 5030.85 | 1.17 | 1.29E-03 | miR-92a-3p | 40541.23 | -1.11 | 1.30E-02 |
| miR-145-3p | 412.67 | 1.17 | 1.67E-04 | miR-378i | 111.31 | -1.12 | 7.63E-03 |
| let-7b-3p | 160.00 | 1.15 | 5.21E-04 | miR-660-5p | 54.08 | -1.13 | 1.29E-02 |
| miR-27a-3p | 14092.08 | 1.06 | 2.21E-04 | miR-501-3p | 437.95 | -1.17 | 1.78E-04 |
| miR-1273h-3p | 88.35 | 1.05 | 8.67E-04 | miR-342-3p | 58.31 | -1.22 | 3.78E-02 |
| miR-320d | 240.37 | 1.00 | 3.17E-02 | miR-18a-3p | 86.31 | -1.30 | 2.73E-02 |
| miR-543 | 946.40 | -1.01 | 3.17E-02 | miR-192-5p | 5623.89 | -1.32 | 1.53E-02 |
| miR-381-3p | 390.63 | -1.08 | 3.15E-02 | miR-511-5p | 478.87 | -1.54 | 1.54E-03 |
| miR-335-3p | 76.99 | -1.08 | 7.59E-03 | miR-182-5p | 1348.23 | -1.58 | 8.06E-04 |
| miR-379-5p | 119.34 | -1.12 | 9.51E-03 | miR-200b-3p | 52.43 | -1.60 | 2.77E-03 |
| let-7b-5p | 27141.91 | -1.15 | 2.18E-02 | miR-200c-3p | 79.53 | -1.66 | 2.77E-03 |
| miR-495-3p | 91.14 | -1.17 | 8.16E-03 | miR-500a-3p | 76.65 | -1.67 | 1.78E-04 |
| miR-215-5p | 423.71 | -1.23 | 2.65E-02 | miR-1-3p | 1044.60 | -1.71 | 1.43E-02 |
| miR-199a-5p | 337.95 | -1.26 | 3.29E-05 | miR-95-3p | 80.54 | -2.13 | 2.14E-04 |
| miR-127-3p | 122.90 | -1.26 | 1.76E-03 | miR-1246 | 510.98 | -2.41 | 1.78E-04 |
| miR-4732-5p | 148.06 | -1.32 | 4.00E-03 |  |  |  |  |
| miR-654-5p | 60.46 | -1.55 | 1.59E-03 |  |  |  |  |
| miR-493-3p | 101.12 | -1.57 | 4.00E-03 |  |  |  |  |
| miR-432-5p | 424.81 | -1.65 | 2.11E-03 |  |  |  |  |

^†^baseMean: mean expression across all samples

^‡^Log2FC: log2 fold change

^§^padj: DESeq2-adjusted p-value


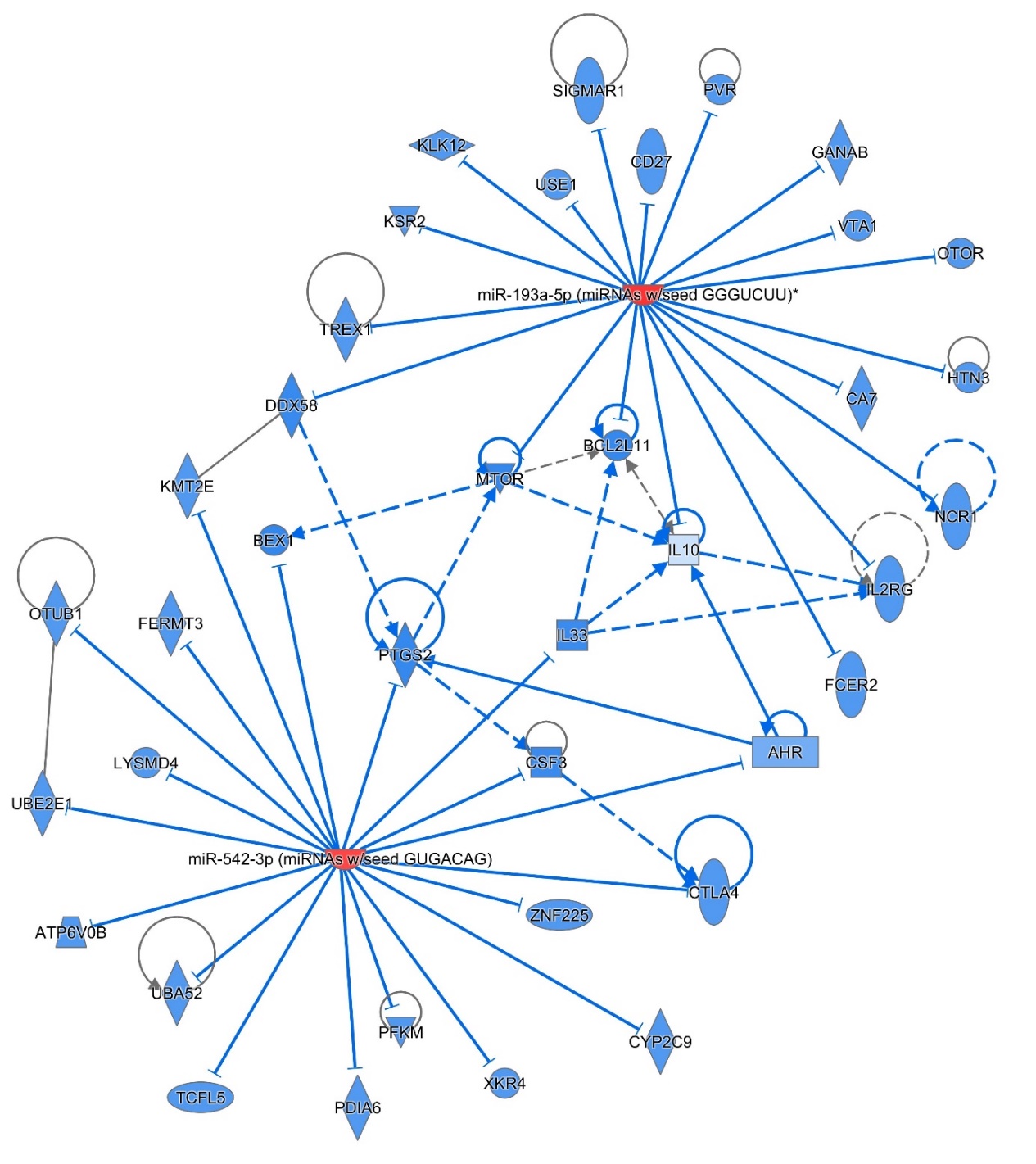


**Figure S1**: Canonical network of the “*cellular and humoral immune response”* generated from the small RNA sequencing data set of the training cohort showing possible downregulated mRNA targets of the technically and additionally validated microRNAs miR-193a-5p and miR-542-3p by reverse transcription quantitative real-time PCR. Data was generated from patients with community-acquired pneumonia (n=12) *vs.* volunteers (n=27). The upregulated miRNAs are shown by red coloring, downregulated target mRNAs are shown in blue. Target filtering was set to “*infectious disease*” and “*experimentally confirmed*” or “*highly predicted*”.

***References***

1. **Shankar-Hari M, Phillips GS, Levy ML, et al.** Developing a New Definition and Assessing New Clinical Criteria for Septic Shock: For the Third International Consensus Definitions for Sepsis and Septic Shock (Sepsis-3). *JAMA*. 2016; 315: 775-87.

2. **Charlson ME, Pompei P, Ales KL, et al.** A new method of classifying prognostic comorbidity in longitudinal studies: development and validation. *J Chronic Dis*. 1987; 40: 373-83.

3. **Lim WS, van der Eerden MM, Laing R, et al.** Defining community acquired pneumonia severity on presentation to hospital: an international derivation and validation study. *Thorax*. 2003; 58: 377-82.

4. **Ronco C.** N-GAL: diagnosing AKI as soon as possible. *Crit Care*. 2007; 11: 173.

5. **Trof RJ, Di Maggio F, Leemreis J, et al.** Biomarkers of acute renal injury and renal failure. *Shock*. 2006; 26: 245-53.

6. **Bone RC.** A New simplified acute physiology score (SAPS II) based on a European/North American multicenter study. *Journal of the American Medical Association*. 1993; 270: 2957-63.

7. **Knaus WA, Draper EA, Wagner DP, et al.** APACHE II: a severity of disease classification system. *Crit Care Med*. 1985; 13: 818-29.

8. **Vincent JL, Moreno R, Takala J, et al.** The SOFA (Sepsis-related Organ Failure Assessment) score to describe organ dysfunction/failure. On behalf of the Working Group on Sepsis-Related Problems of the European Society of Intensive Care Medicine. *Intensive Care Med*. 1996; 22: 707-10.
